# Micro‐Doping of Lithium Ion Battery Cathode Materials ‐ A Performance and Sustainability Case Study of Lithium Nickel Oxide

**DOI:** 10.1002/gch2.202500345

**Published:** 2025-09-08

**Authors:** Isabella D. R. Stephens, Abigail C. Parsons, David Burnett, Peter Slater, Emma Kendrick

**Affiliations:** ^1^ School of Metallurgy & Materials University of Birmingham Elms Road Birmingham West Midlands B15 2SE UK; ^2^ The Faraday Institution Quad One Becquerel Avenue, Harwell Campus Didcot Oxfordshire OX11 0RA UK; ^3^ School of Chemistry University of Birmingham Molecular Sciences Building Birmingham West Midlands B15 2GG UK

**Keywords:** batteries, cathode, cobalt‐free, design, lithium nickel oxide

## Abstract

High‐nickel lithium‐ion battery cathode materials are increasingly favored for their superior energy density but face challenges related to toxicity, cost, and critical material supply. This study assesses the current state of play in commercial cathodes, and presents a screening of the literature micro‐dopants for LiNiO_2_ (LNO), aiming to identify excellent electrochemical performance without compromising affordability or safety. Literature examples of tungsten, niobium, and zirconium doped cathode material all showed good performance, but are considered risky for further consideration due to the high costs and increased supply risk with these materials. A lithium excess sulfate doped material exhibited the best balance of sustainability and performance, delivering improved capacity retention and low raw material cost, with the only compromise of a very slightly elevated supply risk. The study highlights the trade‐offs between performance metrics and sustainability considerations, offering a framework for more commercially viable cathode design.

## Graphical abstract

This study presents a screening of the literature micro‐dopants for LiNiO_2_ (LNO), aiming to identify excellent electrochemical performance without compromising affordability or safety.

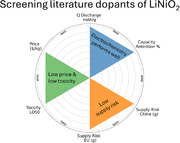

## 1 Introduction

In order to mitigate the increasingly extreme effects of anthropogenic climate change, the world must stop burning fossil fuels.^[^ ^1^, ^2^, ^3^ ^]^ In conjunction with renewable energy, energy storage is a crucial component to decarbonising the grid as well as the transport sector, and the dominant technology is lithium ion batteries (LIBs). According to the International Energy Agency,^[^ ^4^ ^]^ 223 signatories have committed to a Zero‐Emission Vehicles (ZEVs) declaration, calling for all new cars and vans globally to be ZEVs by 2040. This is already greatly increasing the demand for LIBs. Many emerging battery chemistries offer significant promise but lack the performance of LIB. Very few are drop‐in replacements using existing infrastructure. Therefore, LIB will remain dominant forms of energy storage in transport for the foreseeable future.

### 1.1 Commercial Cathode Material History

The first LIB was commercialised by Sony in 1991,^[^ ^5^ ^]^ relying on the reversible exchange of lithium ions (Li^+^) between a graphite (Li_*x*_C_6_) anode and layered LiCoO_2_ (LCO), with the Co^3 + /4 +^ redox couple facilitating energy storage. LCO is a layered transition metal oxide, with a hexagonal α‐NaFeO_2_ structure.^[^ ^6^, ^7^ ^]^ The *O*3 type structure has three sheets of CoO_2_ per unit cell, with lithium ions occupying octahedral sites, and the oxygen stacked in an ABCABC arrangement along the *c* axis.^[^ ^8^ ^]^ As lithium is extracted from LCO, the CoO_2_ sheets can slide over one another, rearranging the structure into a P3 type or O1 type at room temperature. LCO has a high average voltage of 3.79 V vs Li^+^/Li^[^ ^9^ ^]^ and a theoretical capacity of 274 mAh g^−1^. However, the useable capacity is around 140 mAh g^−1^,^[^ ^10^ ^]^ with a high average voltage of ∼3.8 V vs Li^+^/Li, as only around 0.5 mol of Li can be extracted without the structure collapsing. This arises from instabilities associated with the Co^3 + /4 +^ electron arragement. When Co^3 +^ is oxidised, electrons are removed from the t_2_g band which overlaps with the O^2 −^ 2p band. Therefore, deep Li extraction beyond 0.5 mole can oxidise O^2 −^, which increasingly destroys the octahedral CoO_2_ framework and limits the useable capacity. LCO systems however initially dominated the worldwide adoption of LIBs.

LCO is a good example of a complex material with multiple supply chain concerns and constraints. Cobalt is considered a “critical mineral” by the EU,^[^ ^11^, ^12^ ^]^ meaning it has high economic vulnerability, global supply risk and with low substitution ability. Cobalt is also significantly more expensive than other transition metals,^[^ ^13^ ^]^ even before the move to global electrification. Furthermore, more than half of the cobalt mined in the world comes from the Democratic Republic of Congo (DRC),^[^ ^14^ ^]^ with an estimated 15–20%^[^ ^15^, ^16^ ^]^ of that production being extracted by “artisanal miners” on a small scale. This has major ethical implications and local environmental contamination to water supplies, food sources and wildlife.^[^ ^17^ ^]^ For these reasons of ethics, criticality, cost and lower capacity, there is much literature around trying to use less cobalt in LIBs, with market moving to higher and higher nickel content.

LiNiO_2_ (LNO) has also been considered as a possible cathode. It was first synthesised in 1954,^[^ ^18^ ^]^ but LNO was originally dismissed as a lithium‐ion battery cathode, due to safety reasons because of the exothermic collapse of the de‐lithiated Li_*x*_NiO_2_ structure.^[^ ^19^, ^20^ ^]^ LNO has a similar theoretical specific capacity as LCO, at 274 mAh g^−1^, based on the redox couple Ni^3 +^/Ni^4 +^. The nickel content drives increased specific capacity and voltage compared to LCO as the redox couple removes only e_*g*_ band electrons during Li extraction. As this does not overlap with the O^2 −^ 2p fermi level, structural collapse on deep Li removal is mitigated.

**Figure 1:**
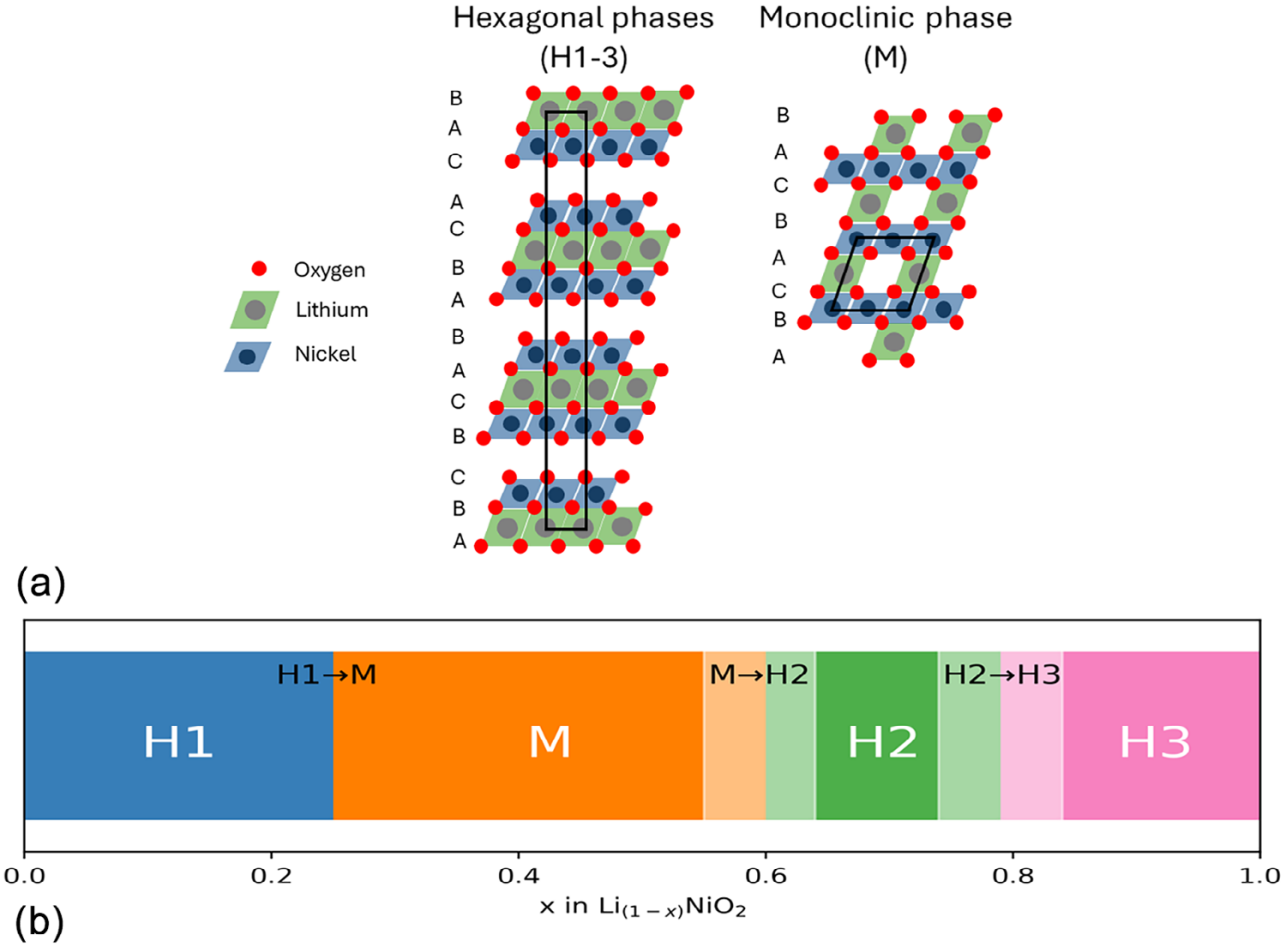
a) Crystal structure representations of the hexagonal and monoclinic phase of LNO.^[^ ^31^, ^32^ ^]^ b) Depiction of phase regions of LiNi_1 − *x*_O_2_ as lithium is removed from the material. The white rectangles represent the slight discrepancy in the literature as to the value at which the phase transition takes place.^[^ ^21^, ^23^, ^24^, ^33^, ^34^ ^]^

As lithium is extracted from the LNO structure, the material undergoes multiple phase transitions which are attributed to the electrostatic interactions of the ionic bonds stabilising the structure. This is described in the literature as transitions from hexagonal (H) and monoclinic (M) phases. As lithium is removed from the LNO structure the transitions go from H1 → M → H2 → H3, which all possess an *O*3 sequence.^[^ ^21^, ^22^, ^23^ ^]^ These phase transitions are depicted in **Figure** **1**. The H2 → H3 transition occurs above ∼4.1 V, and is attributed to the abrupt shrinkage on the c‐axis in the unit cell as well as O_2_ gas evolution, resulting in detrimental anisotropic lattice volume changes.^[^ ^24^ ^]^ This transition is particularly detrimental as it induces significant strain in the material,^[^ ^20^, ^25^ ^]^ and collapse of the structure and capacity. This can cause the cathode material particles to crack,^[^ ^26^ ^]^ which can expose fresh material to side reactions.^[^ ^21^ ^]^

LNO also suffers from a phenomenon called cation mixing. The ionic radius of Li^+^ (0.76 Å) is close to that of Ni^2 +^ (0.69 Å), and hence there can be mixing on the crystallographic 3*b* site, where Ni^2 +^ is expected to be found.^[^ ^27^, ^28^ ^]^ This mixing minimises Coulomb energy and ensures charge neutrality in the structure,^[^ ^29^ ^]^ and causes little dimensional mismatch compared to the equivalent in LCO.^[^ ^18^ ^]^ Partial occupation of these Ni^2 +^ lattice sites by Li^+^ blocks the pathway of lithium diffusion,^[^ ^28^, ^30^ ^]^ and correlates to rapid capacity fade. For all of these reasons, LNO has often been disregarded as a potential LIB cathode material.

Recently, there has been a trend toward high performance cathodes based on high Ni content layered materials. These are structurally akin to LCO/LNO parent phase, using single site multi dopant approaches, such as LiNi_*x*_Mn_*y*_Co_1 − *x* − *y*_O_2_ (NMC) and LiNi_*x*_Mn_*y*_Al_1 − *x* − *y*_O_2_ (NCA). LiNi_0.9_Mn_0.05_Co_0.05_O_2_ (NMC955) represents a state of the art NMC material, with Ni almost entirely occupying the transition metal site. This improves the performance, and as Ni is non‐critical element that is cheaper and more abundant than Co, also reduced the price.

### 1.2 Micro‐Doping Performance in Literature

Commercial cathode materials are tending to greater percentages of nickel, as manufactured cells have gone from NMC111 to NMC622 and more recently NMC811 and NMC955. Hence, investigating the effect of micro‐doping onto the parent phase of pure LNO is an excellent starting point for assessing impact upon performance and sustainability aspects.

Crucially, LNO needs to be stabilized to avoid detrimental collapse of crystal structure on cycling, in order to be a real consideration to become a commercially available material. This can be measured partly by the capacity retention after cycling. **Table** **1** summarises the literature available for LNO doped with single elements and tested for capacity retention.

**Table 1:** Reported electrochemical performance for the different dopants and the relevant voltage ranges and currents used, along with the pure material synthesised and tested in the same way.

Dopant	Material	Q Discharge [mAh g^−1^]	Formation Current	Capacity Retention %	Current and number of cycles	Voltage Range vs Li/Li^+^	Refs.
2 % Mo	Mo‐LNO	240	10mA g^−1^	52	10mA g^−1^ 100 cycles	2.7–4.3V	^[^ ^23^ ^]^
	PureSS‐LNO	199		41			
1 % Nb	LNO‐Nb1	214.08	0.1C	85.78	0.5C 200 cycles	3–4.3V	^[^ ^36^ ^]^
2 % Nb	LNO‐Nb2	205.38		92.62			
0.5 % Nb	LNO‐Nb0.5	241.89		63.54			
	Pure‐LNO	233.69		60.11			
0.4 % Zr	Zr‐doped LNO	246.5	18mA g^−1^ (0.1C)	81	90mA g^−1^ (0.5C) 100 cycles	2.7–4.3V	^[^ ^37^ ^]^
	Pure LNO	247.5		74			^[^ ^37^ ^]^
3 % Y	3Y‐LNO coat	225	18mA g^−1^ (0.1C)	63.1	90mA g^−1^ (0.5C) 100 cycles	2.8–4.3V	^[^ ^38^ ^]^
	PureLNO	225		51.8			
1 % W	W1LNO	242.7	0.1C	90.3	90mA g^−1^ (0.5C) 100 cycles	2.7–4.3V	^[^ ^39^ ^]^
1.5 % W	W1.5LNO	236.1		93.5			
2 % W	W2LNO	225.9		95.5			
	PureLNO	247.5		73.7			
2.5 % S	S‐LRNO	245	25mA g^−1^	83.4	100 mA g^−1^ 100 cycles	2–4.3V	^[^ ^35^ ^]^
	LNO	223	25mA g^−1^	80.5		2–4.3V	

Focus has thus far been mostly on doping cations of a similar octahedral size to the Ni onto the Ni B site. The sulfate material relies on the less used method of poly‐anion doping, introducing the ${\mathrm{SO}}_{4}^{2-}$ ion, which has the S^6 +^ cation. Dong et al. report that the sulfate is located on the nickel site, with a low number of oxygen vacancies. The energetics of the structure is comparable to other high valence dopants, including W and Mo.^[^ ^35^ ^]^

### 1.3 Toxicity, Supply Risk, and Price

A key sustainability challenge for lithium‐ion batteries is the toxicity of precursor and active materials. Murdock *et al.* ^[^ ^40^ ^]^ identified occupational exposure risks as a concern, noting that nickel is more toxic than cobalt. The median lethal dose (LD50) for nickel sulfate hexahydrate, a common precursor, is reported at 362 mg/kg,^[^ ^41^ ^]^ compared to 582 mg/kg^[^ ^42^ ^]^ for cobalt sulfate heptahydrate, indicating significantly higher toxicity for Ni systems. As industry trends shift cathode formulations toward nickel‐rich compositions, particularly in Ni‐rich NMC systems, revisiting the LNO parent phase presents an opportunity to reassess material choices through a sustainability lens.

Rising global battery demand also amplifies pressure on critical raw materials. By 2040, demand for lithium, nickel, cobalt, and graphite is projected to increase between 6 and 13 times.^[^ ^43^ ^]^ This sharp growth exposes manufacturers to volatility in pricing and risks associated with material scarcity and supply chain instability. In response, entities like the European Union (EU) and China, two key players in battery production, have established criticality assessments. These frameworks identify “critical” minerals by their high economic vulnerability, high global supply risks, and low substitution ability.^[^ ^12^, ^44^ ^]^

The priorities for a lithium ion battery cathode material have vastly changed since LNO was first synthesised in 1954, and even since nickelate chemistries became a focus from the 1990s. Whilst extra capacity is important, a defining factor of success is now price. Lithium‐ion battery pack prices have dropped 20% between 2023 and 2024, due to a cell manufacturing overcapacity in China, and the adoption of lower cost LFP batteries among other reasons.^[^ ^45^ ^]^ Globally, from 2021 to 2023 LFP increased its share of battery capacity of electric vehicle sales from 28% to 40% according to the International Energy Agency.^[^ ^46^ ^]^ Within China, these numbers were even more extreme with LFP going from 52 % in 2021 to 67 % in 2023. In November 2024 reports showed LFP prismatic cells at 48.5 $/kWh compared to NMC at 62.4 $/kWh.^[^ ^47^ ^]^ Whilst metals prices do fluctuate, overall CAM price is a key driver of success in commercial materials. LFP cells are also safer, and less likely to reach full thermal runaway than high nickel cells,^[^ ^48^, ^49^ ^]^ with thermal propagation reported in NMC811 as five times faster than in LFP.^[^ ^50^ ^]^

Micro‐doping of lithium ion battery materials, where larger element atoms beyond the first row transition metals are stoichiometrically added in strategic (0.01–0.05 mole) quantities, has become common across layered, lithium excess and disordered rock salt type LIB cathode materials.^[^ ^51^ ^]^ These dopants can enhance structural integrity, capacity retention, and voltage. However, the introduction of such elements also brings potential drawbacks in real‐world applications, particularly in terms of cost, availability, and toxicity. These trade‐offs may ultimately outweigh the benefits observed under laboratory conditions and must therefore be also evaluated.

There are many examples of dopants within the high nickel NMC cathodes in the literature. In order to focus on the impact of the dopant, rather than NMC blend also impacting, this initial study only includes doped literature of pure LNO. It is acknowledged that many additional dopants show promise in NMC chemistry, including Ti,^[^ ^52^ ^]^ Ta, Si, Mg,^[^ ^53^ ^]^ Cu,^[^ ^54^ ^]^ and Li_3_BO_3_.^[^ ^55^ ^]^

To that end, this study compiled comparative data on price and toxicity for literature micro‐dopants in **Table** **2** used in LNO. The literature performance and exact testing regime of all materials is summarised in Table 1.

**Table 2:** Dopants in literature and their supply risk in both China^[^ ^56^ ^]^ and EU,^[^ ^11^ ^]^ prices and toxicity LD50.

Element	EU supply	China supply	Material used	Price	LD50 Oral	Literature Usage
	risk^[^ ^11^ ^]^	risk^[^ ^56^ ^]^		($/kg)	mg/kg	
Ni	0.5	1.52	Nickel Sulfate Hexahydrate	3.6^[^ ^57^ ^]^	362 ^[^ ^41^ ^]^	
Li	1.9	4.66	Lithium Carbonate	10.6^[^ ^57^ ^]^	525 ^[^ ^58^ ^]^	
Mo	0.8	0.84	Molybdenum Trioxide	37.5 ^[^ ^59^ ^]^	2689^[^ ^60^ ^]^	In LNO^[^ ^23^ ^]^
Nb	4.4	5.25	Ammonium niobate	180 ^[^ ^61^ ^]^	not toxic ^[^ ^62^ ^]^	In LNO^[^ ^36^ ^]^
			oxalate hydrate			
Zr	0.8	5.66	Zirconium Nitrate	27.9 ^[^ ^63^ ^]^	2290 ^[^ ^64^ ^]^	In LNO^[^ ^37^ ^]^
Y	3.5	1.37	Yttrium Oxide	2.7 ^[^ ^65^ ^]^	not toxic ^[^ ^66^ ^]^	Surface coating LNO^[^ ^38^ ^]^
W	1.2	0.7	Tungsten Oxide	79.2 ^[^ ^67^ ^]^	2000 ^[^ ^68^ ^]^	In LNO^[^ ^39^, ^69^ ^]^
S	0.3	0.35	Ammonium Sulfate	0.15 ^[^ ^70^ ^]^	2000 ^[^ ^71^ ^]^	In LNO^[^ ^35^ ^]^

In this study, we quantify sustainability aspects of price, toxicity, energy density and supply risk of nickel and cobalt based commercial cathodes to understand the current state of play. Then we investigate the literature available for micro‐doped lithium‐ion cathode material LNO, to evaluate the impact of dopants on material performance. This work aims to establish a sustainability‐first framework for micro‐doping strategies, to guide future research and development in lithium‐ion battery cathode design.

## 2 Experimental Section

### 2.1 Weighted Criticality, Toxicity, and Price

Cathodes are a combination of elements often assessed for price, supply risk and toxicity individually. To assess these aspects, the relative values were weighted to the molar fraction of each element. This method was used with regards to weighted criticality in Stephens *et al.* ^[^ ^72^ ^]^.

#### 2.1.1 Toxicity

One way of measuring toxicity is the median lethal dose (LD50), the dose of substance required to kill half of the members of a tested population within a specified time frame.^[^ ^73^ ^]^ Rats were often used as the test population. The smaller a reported value of LD50, the more lethal the substance. Compounds with an LD50 of greater than 2000 mg/kg are considered to have low toxicity.^[^ ^74^ ^]^ LD50 is a widely available value from safety datasheets when purchasing chemicals, and so offers a very accessible way to compare toxicity.

Values of LD50 are not widely available for CAM. Since workers are likely to come into contact with the starting materials, their toxicity was used as a proxy for overall CAM toxicity.

Values for LD50 reported above the threshold considered as low toxicity were capped at 2000 to avoid skewing results, and the molar fraction of oxygen ignored to consider only the toxicity of included metals.

#### 2.1.2 Supply Risk

A critical minerals assessment has been released every three years in the EU since 2011.^[^ ^75^ ^]^ This measured the supply risk and economic importance of minerals. Those deemed “critical” represented materials that modern societies rely on, with high economic vulnerability, global supply risk and with low substitution ability. Literature for this work focused on just supply risk as the only economic importance, which depends on manufacturing sector and substitution index of a raw material.^[^ ^76^ ^]^ Here we looked only at batteries, with the substitution assessed by comparative analysis of varying cathode chemistry.

The EU assessed 70 candidate materials in 2023,^[^ ^11^ ^]^ grouping Yttrium into the heavy rare earth elements (HREEs). A very similar methodology was used to assess China's criticality by Yan *et al.* ^[^ ^56^ ^]^ The values are listed in **Table** **A1**. These two locations consist of the most comprehensive sources as the EU database comprises significant information on criticality, whereas the literature report from China relates directly to the world's largest battery manufacturing country.

**Table 3:** commercial cathode CAM price, including the calculated one for LNO from ^[^ ^4^ ^]^, and the weighted material price, LD50 toxicity, supply risk. Energy density is calculated as in Equation 5, and the average voltage for LNO is assumed to be the same as NMC955, and NMC532 and NMC111 the same as NMC622.

Material	CAM Price ($/kg)	Capacity (mAh g^−1^)^[^ ^9^ ^]^	Average Voltage (V)^[^ ^9^ ^]^	Energy Density (Wh/g)	Weighted as described in 2.1			
					Price ($/kg)	LD50 Oral	Supply Risk (g)	
							EU	China
LNO	21.75	270	3.73	1.01	2.92	254.99	0.44	1.25
NMC811	19.6^[^ ^57^ ^]^	213.6	3.7	0.79	2.76	291.66	0.61	1.43
NMC622	16.6^[^ ^57^ ^]^	190.9	3.7	0.71	2.61	328.59	0.79	1.62
LCO	18.6^[^ ^57^ ^]^	140	3.79	0.53	2.98	387.75	1.82	3.63
NMC532	15.5^[^ ^57^ ^]^	155	3.7	0.57	2.44	352.36	0.84	1.56
NMC111	15.25	150	3.7	0.56	2.40	378.14	1.04	1.87
NMC955	20.75	230	3.73	0.86	2.84	273.29	0.52	1.34
NCA	19.8^[^ ^57^ ^]^	202	3.69	0.75	2.87	338.65	0.66	1.59

**Table 4:** LNO summary of fraction contributed by Li and Ni.

		Percentage	
	Total value	Li	Ni
Toxicity LD50 mg/kg	255	0.15	0.85
Supply risk EU	0.436	0.31	0.69
Supply Risk China	1.245	0.27	0.73
Price	2.92	0.26	0.74
Molar mass	97.6	0.07	0.60

**Table 5:** Calculation of values for supply chain, toxicity and price, included with capacity retention and Q discharge from the literature.

Reference	Name	Formula	Supply Risk		Toxicity	Q Discharge	Capacity	Price
			EU	China		[mAh g^−1^]	Retention %	$/kg
^[^ ^23^ ^]^	Mo‐LNO	Li_1.03_Mo_0.02_Ni_0.95_O_2_	0.444	1.236	287	240	52	3.60
	Price‐PureSS‐LNO	LiNiO_2_	0.436	1.245	255	199	41	2.92
^[^ ^36^ ^]^	LNO‐Nb1	LiNi_0.99_Nb_0.01_O_2_	0.473	1.274	271	214.08	85.78	4.59
	LNO‐Nb2	LiNi_0.98_Nb_0.02_O_2_	0.510	1.302	287	205.38	92.62	6.26
	LNO‐Nb0.5	LiNi_0.995_Nb_0.005_O_2_	0.454	1.259	263	241.89	63.54	3.76
	Hao‐Pure‐LNO	LiNiO_2_	0.436	1.245	255	233.69	60.11	2.92
^[^ ^37^ ^]^	Zr‐dope LNO	Li_1.06_Ni_0.937_Zr_0.04_O_2_	0.453	1.414	317	246.5	81	3.86
	Yoon‐Pure LNO	LiNiO_2_	0.436	1.245	255	247.5	74	2.92
^[^ ^38^ ^]^	3Y‐LNO coat	LiNiO_2_Y_0.03_	0.517	1.249	301	225	63.1	2.91
	Zhang‐PureLNO	LiNiO_2_	0.436	1.245	255	225	51.8	2.92
^[^ ^39^ ^]^	W1LNO	LiNi_0.99_W_0.01_O_2_	0.450	1.234	287	242.7	90.3	4.33
	W1.5LNO	LiNi_0.985_W_0.015_O_2_	0.456	1.228	302	236.1	93.5	5.03
	W2LNO	LiNi_0.98_W_0.02_O_2_	0.463	1.222	318	225.9	95.5	5.71
	Ryu‐PureLNO	LiNiO_2_	0.436	1.245	255	247.5	73.7	2.92
^[^ ^35^ ^]^	S‐LRNO	Li_1.1_Ni_0.875_S_0.025_O_2_	0.441	1.243	261	245	83.4	2.90
	Dong‐LNO	LiNiO_2_	0.436	1.245	255	223	80.5	2.92

#### 2.1.3 Price

Price for battery materials, both raw and cathode active material (CAM), is reported in $/kg. Raw materials sold online in different units were converted to $/kg, using the exchange rates of £ 1 to US$ 1.26, and 0.14 CNY to US$ 1. For Zirconium Nitrate where many prices were available online,^[^ ^63^ ^]^ the industrial grade material was estimated at 200 CNY.

A clear limitation is that prices can fluctuate. This methodology is based on available data at publication, but will need updating regularly to reflect real world price situations.

#### 2.1.4 Calculations

The supply risk for each chemistry was calculated for 1 g of CAM using Equation 1.

(1) $$\begin{array}{cc} \text{Weighted Supply Risk} & =\sum_{n}n\;\text{Mass fraction of component}\\ & \;\times \text{Supply risk} \end{array}$$

for over *n* raw material components contained in 1g of CAM.

The same principle can be applied to raw material cost, and toxicity values, giving Equations 2 and 3:

(2) $$\begin{array}{c} \text{Weighted Raw Material Price}\frac{\$}{\text{kg}}=\\ \sum_{n}n\;\text{Mass fraction of component}\times \text{Raw material price}\frac{\$}{\text{kg}} \end{array}$$

(3) $$\begin{array}{cc} \text{Weighted Toxicity} & =\sum_{n}n\;\text{Mass fraction of metal component}\\ & \;\times \text{Raw Material LD50} \end{array}$$

The value for oxygen for supply risk, price and toxicity is considered to be zero.

**Figure 2:**
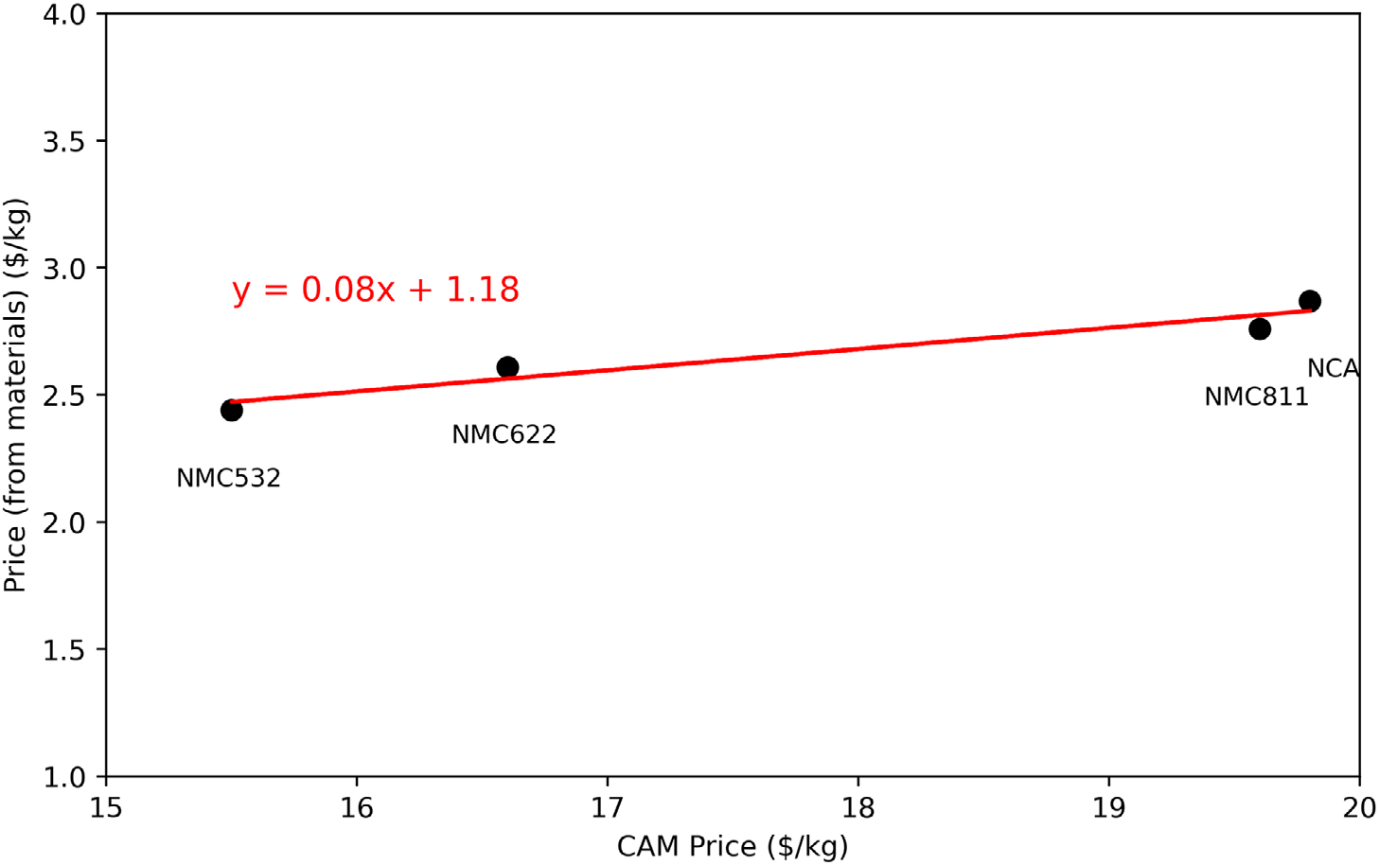
Plot of price calculated from molar amounts of cathode material starting price, and the CAM cost as of January 2025.

In order to predict a commercial price for LNO, the raw material price (as calculated from the raw materials and mass fraction described in 2.1.3) was plotted against the CAM price (**Figure** **2**).

This gives the following Equation 4 which results in an estimate of 21.75 $/kg for LNO. NMC111 and NMC955 were also calculated.

(4) $$\text{CAM Price}\left(\$/\text{kg}\right)=\frac{\text{Material Price}(\$/\text{kg})-1.18}{0.08}$$

Energy density is calculated from the specific capacity and average voltage as in Equation 5.

(5) $$\begin{array}{cc} \text{Energy Density (Wh/g)} & =\text{Average Voltage (V)}\\ & \;\times \text{Capacity (Ah/g)} \end{array}$$

## 3 Results and Discussion

### 3.1 Comparing Commercial Cathode Materials

The current state of play was examined. Commercially available high nickel cathode materials studied are NMC811, NMC622, NMC532, NMC111, LiNi_0.8_Co_0.15_Al_0.05_O_2_ (NCA) and next generation LiNi_0.9_Mn_0.05_Co_0.05_O_2_ (NMC955). LCO was also included, as well as theoretical LNO values. The raw values were collected into Table A1 to calculate the values in **Table** **3**.

**Figure 3:**
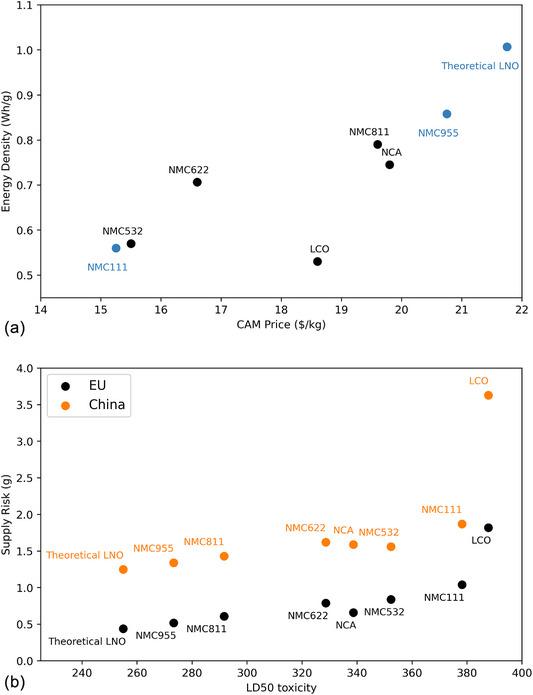
a) Energy density in Wh/g of cathode material plotted against the CAM price. Blue values indicate the CAM price was calculated from Equation 4, and black from literature. b) Weighted supply risk in g for both China^[^ ^56^ ^]^ and the EU^[^ ^11^ ^]^ plotted against weighted LD50 values for toxicity. Lower LD50 values indicate higher toxicity.

The values were plotted in **Figure** **3**a,b. Supply risk showed a strong similarity of trend for both China and the EU, with LCO as a real outlier as being very high supply risk due to the high percentage of cobalt. If the theoretical value of LNO could be achieved for multiple cycles, it would be the lowest supply risk and highest capacity as Nickel has a much lower supply risk in both China and EU than Cobalt.

From these figures and Table 3 it is clear that with an increasing molar fraction of nickel:

- toxicity increases;
- supply risk decreases slightly;
- theoretical energy density increases;
- CAM price increases.

Significantly, researchers often promote the second two factors in LCA assessments, but commonly do not highlight the toxicity increase.

Possible dopants to stabilize high nickel cathodes should be focused on low toxicity and low price, as well as improving the stability of the material. Due to the low supply risk of nickel itself dominating the supply risk of the material, it is less crucial to have the lowest possible supply risk, but ideally low supply risk is also favorable.

The CAM price increase is likely related to the need for particular dry conditions such as heating and dry room conditions, which may significantly increase the energy needed to produce high nickel cathodes.

### 3.2 Pure Lithium Nickel Oxide

**Figure 4:**
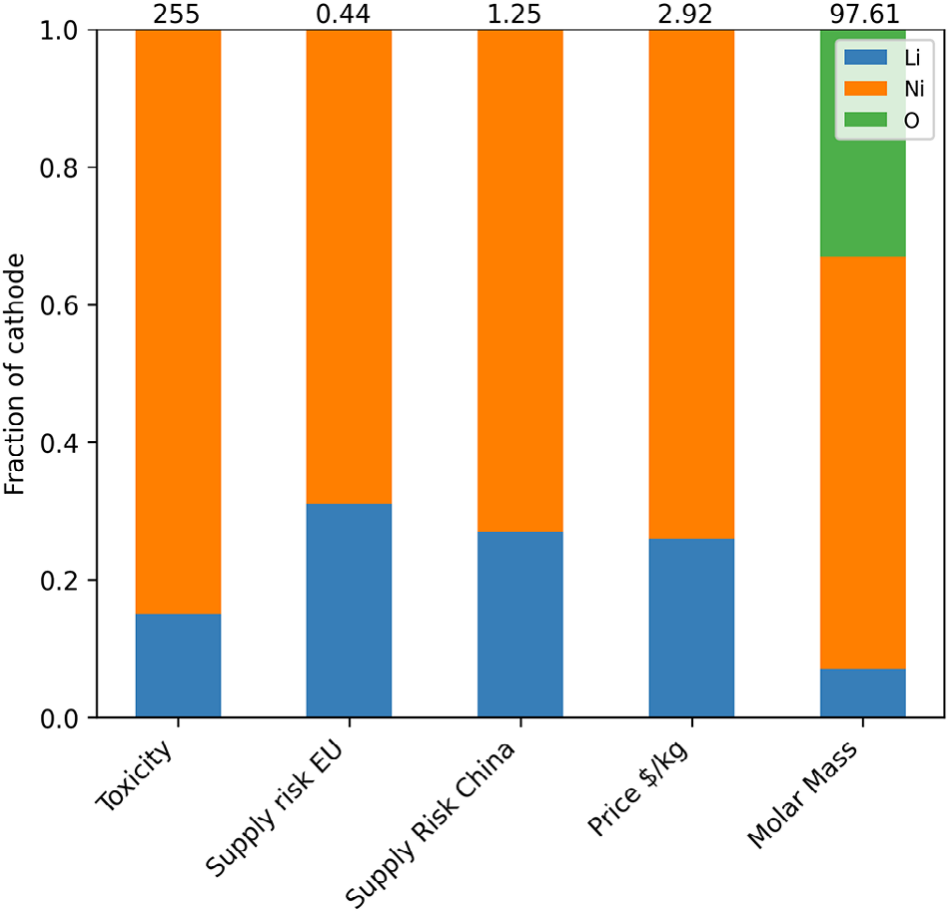
Percentage contributions of the components of LNO to toxicity, supply risk, price and molar mass. Numbers are listed in Table 4.

For pure LNO, the fraction of toxicity, supply risk price and molar mass was detailed in **Table** **4** and displayed in **Figure** **4**. These show the outsized impact that lithium has on particularly price and supply risk.

### 3.3 Analysis of Literature LNO

**Figure 5:**
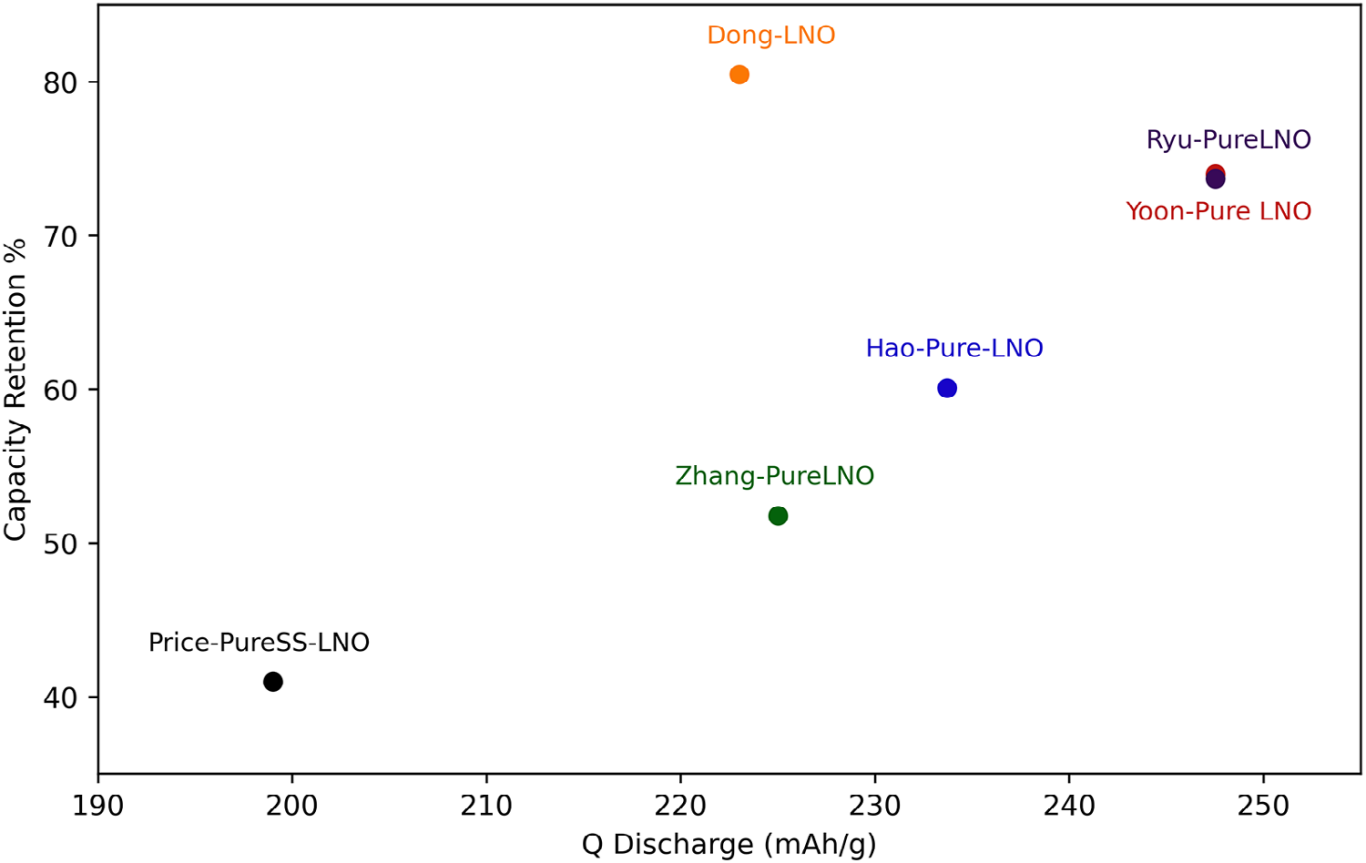
Literature values for capacity retention and initial Q discharge of the pure materials synthesised. Capacity retention is over 100 cycles for all except Hao‐Pure‐LNO which is over 200 cycles.

**Table** **5** details the literature values as well as calculated values that will allow comparability of sustainability. As the pure LNO will all have the same supply risk, price and toxicity, the capacity retention and Q discharge were compared in **Figure** **5**. There is a large discrepancy between both the Q discharge and capacity retention of the pure LNO seen in literature, with two getting close to the theoretical capacity of 270 mAh g^−1^, but all having the typical poor capacity retention after only 100 cycles, or 200 cycles for Hao pure‐LNO.^[^ ^36^ ^]^

**Figure 6:**
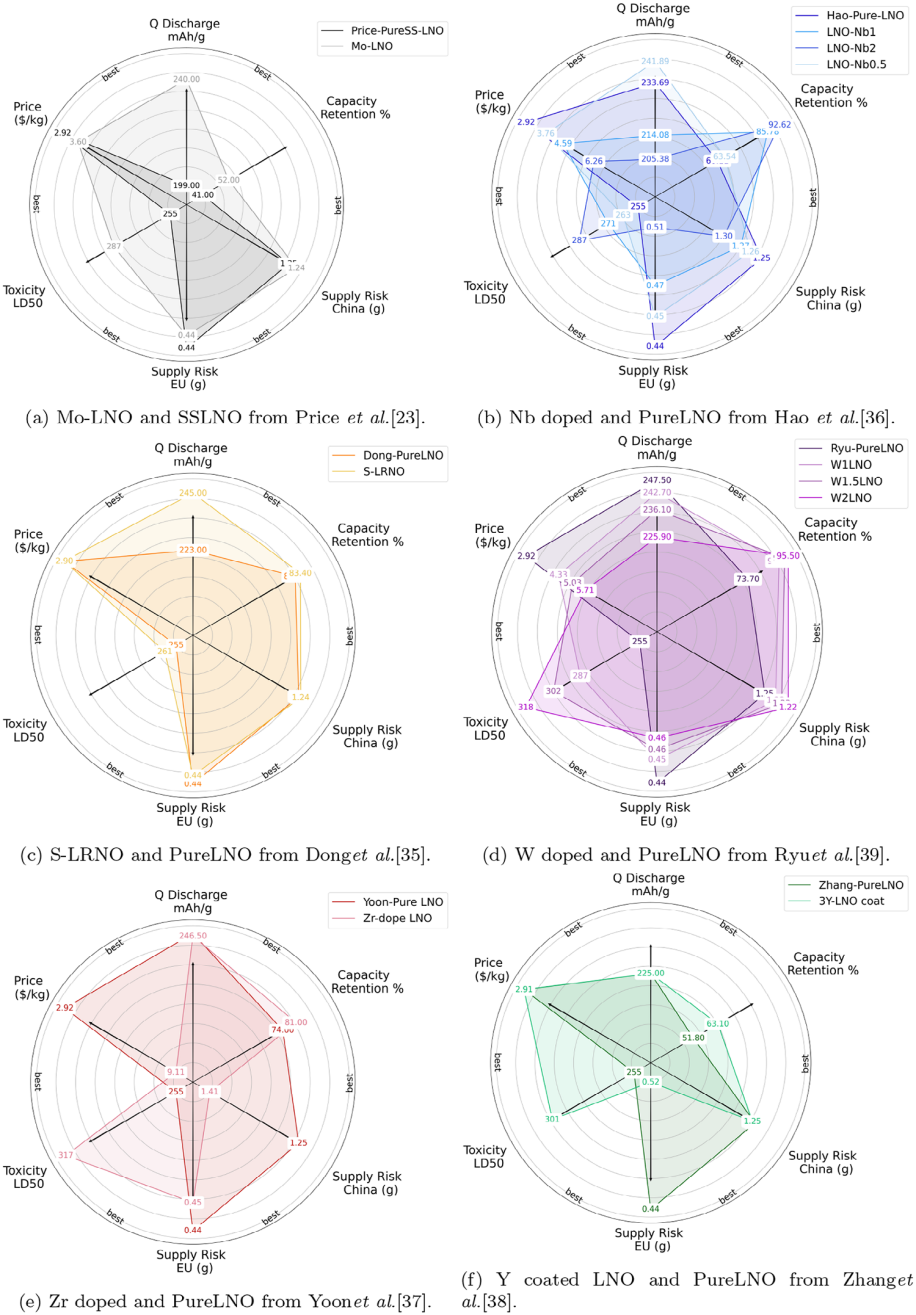
Radar plot of labeled cathodes and their comparative metrics. a) Mo‐LNO and SSLNO from Price et al.^[^ ^23^ ^]^. b) Nb doped and PureLNO from Hao et al.^[^ ^36^ ^]^. c) S‐LRNO and PureLNO from Donget al.^[^ ^35^ ^]^. d) W doped and PureLNO from Ryu et al.^[^ ^39^ ^]^. e) Zr doped and PureLNO from Yoon et al.^[^ ^37^ ^]^. f) Y coated LNO and PureLNO from Zhang et al.^[^ ^38^ ^]^

The doped versions were considered compared to their pure‐LNO made the same way, for consistency. Radar charts showing the supply risk, toxicity, price, Q discharge and capacity retention are shown in **Figure** **6**. Since the numerical scales in the different segments vary substantially from 0.44 to 318, the distance from the origin is scaled relative to the maximum in each respective criteria. This allows all 6 results to be shown simultaneously. The most desirable radar was stretched out to the edges as possible.

Molybdenum increased the Q discharge of the material considerably. However, the capacity retention was still extremely low at 52%, and the price and supply risk were slightly increased by the inclusion of Mo. The toxicity did improve slightly, however this is inevitable when replacing nickel with any less toxic component.

Zirconium, Tungsten and Niobium showed considerable promise with high initial capacity or improved capacity relative to pure.

Niobium doping increased the Q discharge at 0.5% dopant level, and also all Nb doping increases the capacity retention. Increasing the niobium molar fraction also decreases the toxicity. However, all inclusions of Nb make the cathode more expensive, and increase the supply risk.

The tungsten doping decreases the capacity relative to the pureLNO. The capacity retention is improved, however, the price and supply risk are increased by adding tungsten. The toxicity is also markedly improved by adding more tungsten, however this is due to tungsten being a heavy element, and hence having a larger molar fraction than other dopants.

The Zr doped showed considerable promise with improved capacity retention, and much improved toxicity. However, the price increase for this material is very large as the starting material used for zirconium is very expensive. The supply risk is also slightly increased.

Sulfate doping increases the Q discharge, and capacity retention slightly. The toxicity is slightly decreased, and the supply risk is also increased a very small amount. Sulfate is the only dopant to decrease the overall price of the material.

The Yttrium coated material exhibited improved capacity retention relative to pure, but low capacity retention at 63.1%. The toxicity for Y coated is also lowered relative to the pure LNO, however there is a significant increase in the supply risk as Y has a very high supply risk in the EU.

Cobalt and Nickel sulfates are considered expensive battery materials at 3.7 and 3.6 $\frac{\$}{\text{kg}}$ respectively.^[^ ^57^ ^]^ Ammonium niobate oxalate hydrate is considerably more expensive at 180 $\frac{\$}{\text{kg}}$, possibly due to small quantities produced of this material. Zirconium Nitrate is 27.9 $\frac{\$}{\text{kg}}$, with some cheaper sources available on line for lower purity. Zirconium is also used in proton exchange membrane water electrolysis, and as technologies such as this ramp up associated materials may also get cheaper. If a much cheaper source of Zirconium and Niobium was available, then these would be the best materials to consider due to the positive effect they have on the capacity of LNO. However, the driver of many battery industry decisions has been cost and price point.

Designing a new cathode material would ideally find the cheapest, least toxic and most energy dense material with low supply risk and good cycle life. However, so far all commercial cathodes have seemingly compromised on one or more of these. The high energy density of NMC materials is a trade off with the expensive and toxic nature of it. The low energy density of LFP is acceptable because the cost of it is extremely cheap.

If just considering raw capacity values and capacity retention, the most promising materials screened here would be either W1LNO or Zr‐dope LNO with initial discharge capacities of 242.7 and 246.5 mAh g^−1^ and capacity retention of 90.3% and 81% respectively. If the price relationship in Equation 4 remained true, a material price for W1LNO of 4.33 $\frac{\$}{\text{kg}}$ would result in a CAM price of 39.4 $\frac{\$}{\text{kg}}$, which would be completely uncompetitive compared to NMC811 at 19.6 $\frac{\$}{\text{kg}}$. The material price of Zr‐dope LNO of 3.86 $\frac{\$}{\text{kg}}$ would similarly give CAM cost of 33.44 $\frac{\$}{\text{kg}}$.

In summary, the most effective compromise material identified in this screening was the sulfate doped LNO, with only a compromise exhibited on the slight increase supply risk. When considering larger scale up, the volatility of components can be an issue in the synthesis of these cathode materials. In all the systems, Li volatility can be an issue,^[^ ^77^ ^]^ and of the dopants proposed S, Mo, and W can also show volatility issues at elevated temperatures. Therefore, control of synthesis temperature and atmosphere is particularly important at larger scales. The volatility of many components (e.g., Li and S) can be increased in the presence of moisture,^[^ ^77^, ^78^ ^]^ so synthesis in inert or dry O_2_ atmospheres should be employed.

This study focuses on low cost and low toxicity, which should serve as the foundational criteria when selecting dopants aimed at enhancing properties such as capacity retention. Prioritizing these factors ensures that advances in the literature remain aligned with commercial viability and practical implementation in battery technologies.

## 4 Conclusion 

This study explored LNO as a model cathode system, incorporating literature values for micro‐dopants to assess their impact on electrochemical performance and sustainability. The literature values for capacity retention and energy density were used to compare with dopant toxicity, raw material cost, and supply risk.

Among the dopants examined, Niobium, Tungsten, and Zirconium showed the most promising electrochemical performance, particularly in terms of capacity retention. However, the high costs and associated supply risks render them commercially unattractive despite its electrochemical advantages. In contrast, the sulfate dopant demonstrated modest yet meaningful enhancements in performance while maintaining favorable sustainability profiles, at a fraction of the cost and criticality risk associated with niobium.

Overall, this work highlights the importance of considering economic and environmental metrics alongside performance when designing next‐generation cathode materials. While dopants such as S may not deliver the highest absolute performance, low toxicity, low cost, and secure supply chains make it more viable for larger‐scale adoption and warrant further investigation. These findings underscore the need for a holistic approach to cathode material development—one that strikes a balance between performance gains and real‐world constraints.

We propose that future research in lithium‐ion cathode doping strategies should adopt a sustainability‐first perspective to ensure that laboratory innovations translate into commercially and ethically viable technologies.

## Conflict of Interest

The authors declare no conflicts of interest.

## Data Availability

The data that support the findings of this study are available in the supplementary material of this article.

## Appendix A

### A.1 Raw Values Tables

**Table A1:** Raw material supply risk, price and LD50 values.

Element	Supply Risk		Raw material used	Price	LD50
	EU^[^ ^11^ ^]^	China^[^ ^56^ ^]^		[$/kg]	Oral
Ni	0.5	1.52	Nickel Sulfate Hexahydrate	3.6^[^ ^57^ ^]^	362 ^[^ ^41^ ^]^
Li	1.9	4.66	Lithium Carbonate	10.6^[^ ^57^ ^]^	525 ^[^ ^58^ ^]^
Co	2.8	5.48	Cobalt Sulfate Heptahydrate	3.7^[^ ^57^ ^]^	582 ^[^ ^42^ ^]^
Mn	1.2	0.57	Manganese Sulfate Monohydrate	0.8^[^ ^57^ ^]^	782 ^[^ ^79^ ^]^
Al	1.2	0.27	Aluminium Hydroxide	0.5 ^[^ ^80^ ^]^	5000 ^[^ ^81^ ^]^
